# A Computational Approach to Characterizing the Impact of Social Influence on Individuals’ Vaccination Decision Making

**DOI:** 10.1371/journal.pone.0060373

**Published:** 2013-04-09

**Authors:** Shang Xia, Jiming Liu

**Affiliations:** Department of Computer Science, Hong Kong Baptist University, Kowloon Tong, Hong Kong S.A.R; National Institute for Public Health and the Environment, The Netherlands

## Abstract

In modeling individuals vaccination decision making, existing studies have typically used the payoff-based (e.g., game-theoretical) approaches that evaluate the risks and benefits of vaccination. In reality, whether an individual takes vaccine or not is also influenced by the decisions of others, i.e., due to the impact of social influence. In this regard, we present a dual-perspective view on individuals decision making that incorporates both the cost analysis of vaccination and the impact of social influence. In doing so, we consider a group of individuals making their vaccination decisions by both minimizing the associated costs and evaluating the decisions of others. We apply social impact theory (SIT) to characterize the impact of social influence with respect to individuals interaction relationships. By doing so, we propose a novel modeling framework that integrates an extended SIT-based characterization of social influence with a game-theoretical analysis of cost minimization. We consider the scenario of voluntary vaccination against an influenza-like disease through a series of simulations. We investigate the steady state of individuals’ decision making, and thus, assess the impact of social influence by evaluating the coverage of vaccination for infectious diseases control. Our simulation results suggest that individuals high conformity to social influence will increase the vaccination coverage if the cost of vaccination is low, and conversely, will decrease it if the cost is high. Interestingly, if individuals are social followers, the resulting vaccination coverage would converge to a certain level, depending on individuals’ initial level of vaccination willingness rather than the associated costs. We conclude that social influence will have an impact on the control of an infectious disease as they can affect the vaccination coverage. In this respect, our work can provide a means for modeling the impact of social influence as well as for estimating the effectiveness of a voluntary vaccination program.

## Introduction

In the control of infectious diseases by voluntary vaccination, individuals decisions on whether or not taking the vaccine will affect the vaccination coverage and, hence, the effectiveness of disease control [1], [2], since the prevention of disease transmission requires the vaccination coverage of a host population to be above the level of herd immunity threshold [3].

Existing studies on individuals vaccination decision making have typically focused on several determinants associated with the risks and benefits of vaccination, including the perceived risk of disease infection [4]–[6], the perceived safety and efficacy of vaccine [7], [8] (e.g., vaccine side-effect rate and the related adverse complications), as well as the social financial costs associated with vaccination and disease infection [9] (e.g., charge of vaccine administration, expenses for infection treatment, and absence from work).

Besides these factors, individuals vaccination decisions are also subjected to the impact of social influence in that an individuals behaviors or opinions are affected by those of others [10]. For example, the social influence on individuals vaccination decisions can come from the interactions among them, such as recommendations given by friends or family members [6], [11], suggestions from health professionals [12], and advices given by trusted colleagues [13]. The effects of social influence on human health related behaviors have long been observed. In the case of 2003 SARS outbreak in China, individuals avoidance behaviors arose as a response to the circulation of short messages about disease outbreaks [14]. As for vaccination, health related newscasts would change individuals perceptions of vaccine safety and efficacy [15]. The attitudes shared among parents would influence the vaccination decisions of their children [16]. In this regard, modeling vaccination decision making should be treated as not merely a process of payoff optimization, but also a process of individuals response to the impact of social influence.

**Figure 1:**
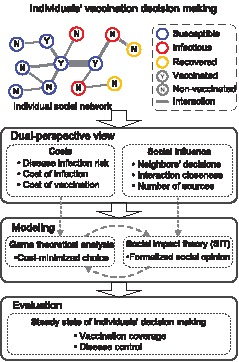
A dual-perspective view on modeling individuals vaccination decision making. We extend the existing game-theoretical approaches by incorporating the impact of social influence. A group of interactive individuals can make decisions by both minimizing the associated costs and evaluating the decisions of others. We utilize social impact theory (SIT) to characterize the impact of social influence on individuals decision making with reference to their interaction relationships. We use a social contact network structure to represent individuals interaction relationships. By doing so, we can investigate the steady state of individuals decision making and examine the impact of social influence on vaccination dynamics and hence disease control, in terms of the vaccination coverage and the size of disease infections, respectively.

In order to better understand individuals vaccination decision making, in this study, we take a dual-perspective view to address both the cost analysis of vaccination decisions and the impact of social influence. As illustrated in Figure 1, we consider a group of individuals that make their vaccination decisions by both minimizing the associated costs and evaluating the decisions of others (i.e., social influence). Specifically, we consider that the social settings of individuals are structured with reference to their interaction relationships (i.e., connected individuals and their social closeness). Therefore, the impact of social influence among them will be heterogeneous with respect to the structure of their interactions. In addition, when individuals interact with those having similar choices, their decisions may be further affirmed; otherwise, their decisions may be weakened [17]. In such a case, social impact theory (SIT) provides a computational approach to characterizing the impact of social influence with respect to individuals interaction relationships [18]. Generally speaking, SIT describes how individuals change their attitudes/decisions in a structured social environment, and further suggests that the strength of the social impact be determined by the characteristics of the source (e.g., various attitudes/decisions), the closeness of their social relationships, and the number of sources holding similar attitudes/decisions [19]. In our current work, we propose a novel modeling framework for describing individuals vaccination decision making by integrating an extended SIT-based characterization of social influence with a game-theoretical analysis of cost minimization. In this model, we use a conformity rate to describe the impact of social influence on vaccination decision making, in terms of individuals tendency of being affected by the social influence of others. Additionally, we represent individuals interaction relationships with reference to a social network structure, in which individuals are heterogeneously connected with different numbers of connected neighbors and the social closeness of their interactions. We parameterize the proposed model with an influenza-like disease as well as a real-world social network.

By carrying out a series of simulations on voluntary vaccination, we examine the steady state of individuals decision making and evaluate the vaccination dynamics as well as the effect of disease control, in terms of vaccination coverage and the resulting disease infection rate, respectively. By doing so, we aim to investigate the interplay of cost minimization and social influence on individuals vaccination decision making, and examine the impacts of different levels of individuals conformity towards the impact of social influence. Furthermore, we provide a new modeling framework that incorporates the impact of social influence for investigating the effectiveness of voluntary vaccination for infectious diseases control.

## Methods

We consider a voluntary vaccination program for controlling an influenza-like infectious disease (e.g., seasonal flu), in which individuals need to decide whether or not to be vaccinated each season based on their perceived risk of disease infection. It is assumed that individuals will have some knowledge about the vaccine and the disease (e.g., acquired from their previous experience and/or from public media and health authorities), and about others vaccination decisions through their social interactions. For such a situation, we construct a computational model that describes how an individual arrives at his/her vaccination decision with respect to the cost analysis of vaccination decisions, and the social influence of others decisions. Based on the constructed model, we aim to investigate the impact of social influence on individuals vaccination decisions as well as on the disease control.

### Vaccination Decision Making

**Figure 2:**
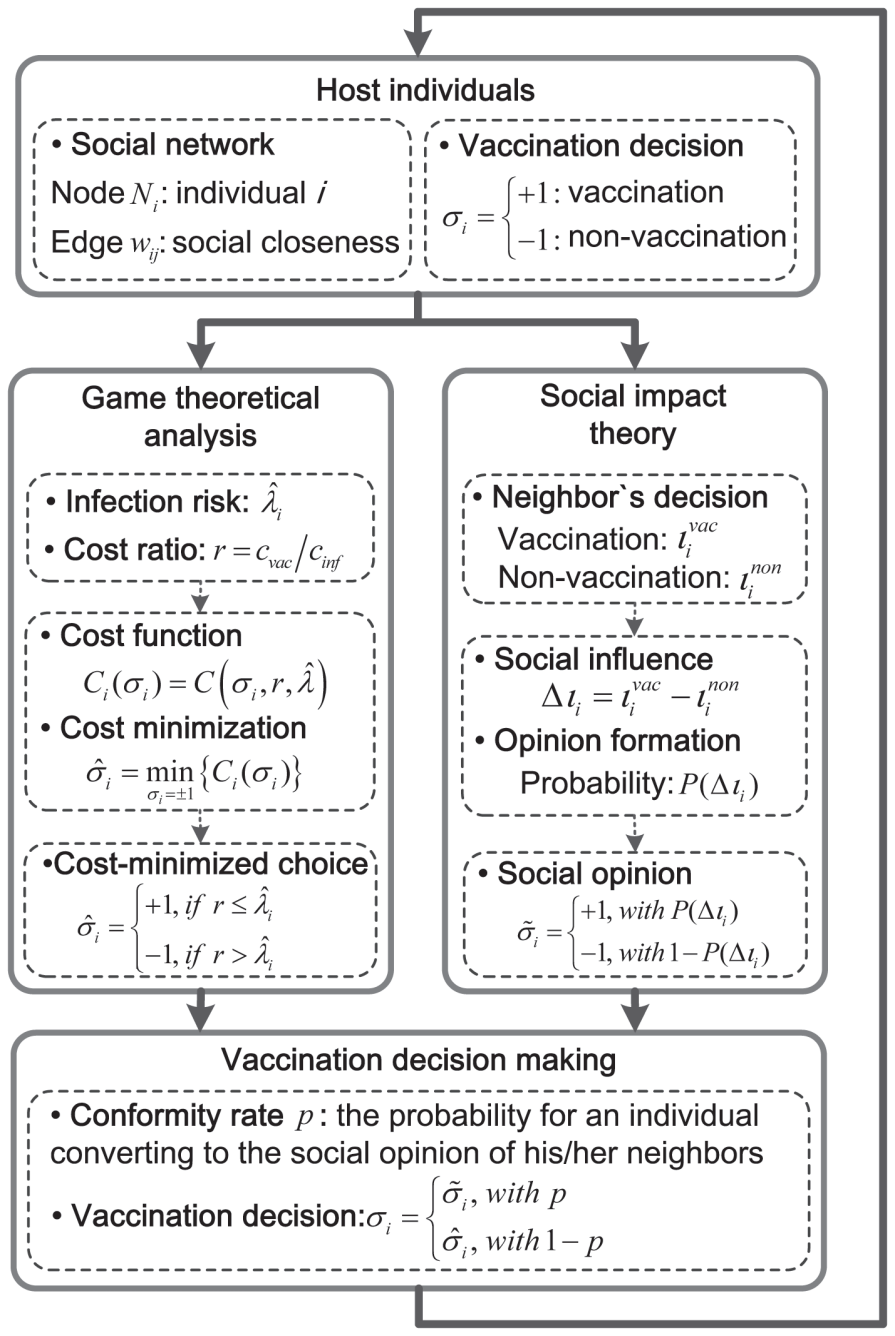
The proposed model of individuals vaccination decision making. We consider an individuals vaccination decision making with respect to (1) cost minimization, and (2) impact of social influence. We construct a game-theoretical model to describe how an individual arrives at a cost-minimized choice (i.e., denoted by 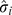) by evaluating the costs of vaccination and infection as well as the risk of disease infection. We utilize social impact theory (SIT) to characterize the formation of a social opinion (i.e., denoted by 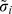) from an individuals connected neighbors based on the social influence of two opposite opinions. Here, 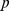 denotes the conformity rate, which is the probability that an individual finally convert to the formalized social opinion, or otherwise follows his/her cost-minimized choice.

We take a dual-perspective view on modeling individuals vaccination decision making that incorporates individuals evaluation of vaccination associated costs as well as the impact of social influence. In doing so, we introduce an individual-based model, as described in Figure 2. In the figure, 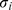 denotes an individual 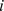
s vaccination decision. There are two possible decisions that an individual can make: 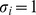 corresponds to an acceptance of vaccination, and 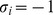 represents a rejection. We utilize a social network to characterize the structure of individuals interactions, in which the nodes correspond to individuals and te edges denote the interaction relationships among them. Each edge has a weight 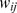, which represents the closeness of interactions between individuals 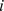 and 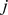.

Individuals can evaluate the costs associated with their decisions and then arrive at their optimal choices by minimizing the costs. Meanwhile, individuals may also convert their decisions due to the impact of social influence (i.e., neighbors vaccination decisions). Thus, individuals vaccination decision making will be modeled here to include two aspects: (1) cost minimization and (2) the impact of social influence. The parameters used for modeling individuals vaccination decision making are summarized in Table 1.

**Table 1:** Parameters used for modeling vaccination decision making.

Symbol	Meaning
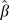	perceived disease transmission rate
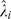	perceived infection risk
*c_inf_*	cost of disease infection
*c_vac_*	cost of vaccination
*r*	cost ratio 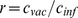
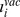	social influence for vaccination
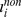	social influence against vaccination
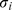	vaccination decision
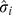	cost-minimized choice
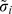	social opinion of connected neighbors
*p*	conformity rate

#### Cost minimization

There are two types of costs associated with an individuals vaccination decision: (1) the cost of vaccination (e.g., the potential risk of vaccine side-effects or the expense of vaccine administration) and (2) the cost of disease infection if not vaccinated (e.g., disease complications, expenses for treatment, or absence from work). We let 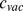 and 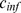 denote the costs associated with vaccination and disease infection, respectively, and use 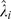 represent the perceived risk of disease infection for individual 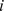. Then, we can introduce a cost function for individual 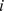 with a decision 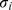, as follows:
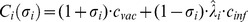
(1)where 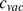 denotes the cost associated with accepting vaccination, and 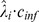 denotes the cost associated with rejecting vaccination.

Next, without loss of generality, we let 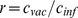 describe the relative ratio of 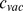 and 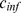. Thus, we can further transform the cost function 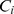 in Eq. 1 into the following:
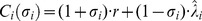
(2)


Here, we assume that individuals can estimate the risk of disease infection based on their perceived disease severity, as reflected in the perceived disease transmission rate, 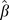, as well as their neighbors vaccination decisions, as represented by 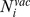 and 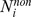 for the numbers of neighbors with the decisions of vaccination or not, respectively. In addition, vaccinated individuals are assumed to be successfully immunized from disease infection and unvaccinated individuals will be possibly infected and thus transmit disease. Therefore, the perceived infection risk, 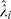, can be computed corresponding to the proportion of unvaccinated neighbors as follows:
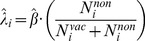
(3)


Based on the above formulation, an individual can arrive at an optimal choice by minimizing the cost function in Eq. 2. In our proposed model, individual 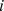 will accept vaccination (i.e., 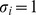) if 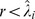, reject vaccination (i.e., 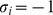) if 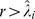, and keep his/her decision unchanged in the previous step if 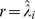. We can write this cost-minimized choice of individual 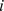, 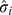, in the following form:
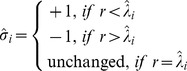
(4)


If all individuals follow the same strategy of minimizing their cost functions, after some iterations of decision making, they will reach a steady state, in which all individuals will have no incentive to change their decisions in the next step.

#### Social influence

In addition to the above-mentioned cost minimization, an individual may affect by those decisions of others, i.e., due to the impact of social influence [18], and then convert the cost-based choice to the social opinion of his/her neighbors. According to social impact theory (SIT) [19], [20], the strength of such a social influence will be subjected to the structure of individuals interactions, e.g., the types of opinions (i.e., acceptance or rejection 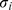), interaction relationships (i.e., social closeness 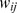), and the number of opinion sources (i.e., the numbers of vaccinated and unvaccinated neighbors, 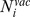 and 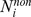, respectively). In our social network, for individual 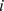, the strengths of social influence for two opposite opinions (i.e., vaccination acceptance and rejection), described by 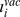 and 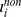, can be accordingly computed based on [19] as follows:
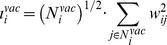
(5)

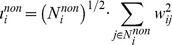
(6)


We use 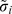 to denote the formalized social opinion resulting from the social influence of individual 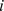
s neighbors. As a modification of the standard SIT definition (where 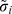 corresponds to the opinion with the larger strength of social influence), 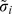 being either acceptance or rejection of vaccination will be determined by comparing the influences of two opposite opinions. We let 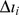 denote the discrepancy between 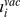 and 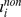. Then, we normalize 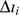 as follows:
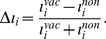
(7)


**Figure 3:**
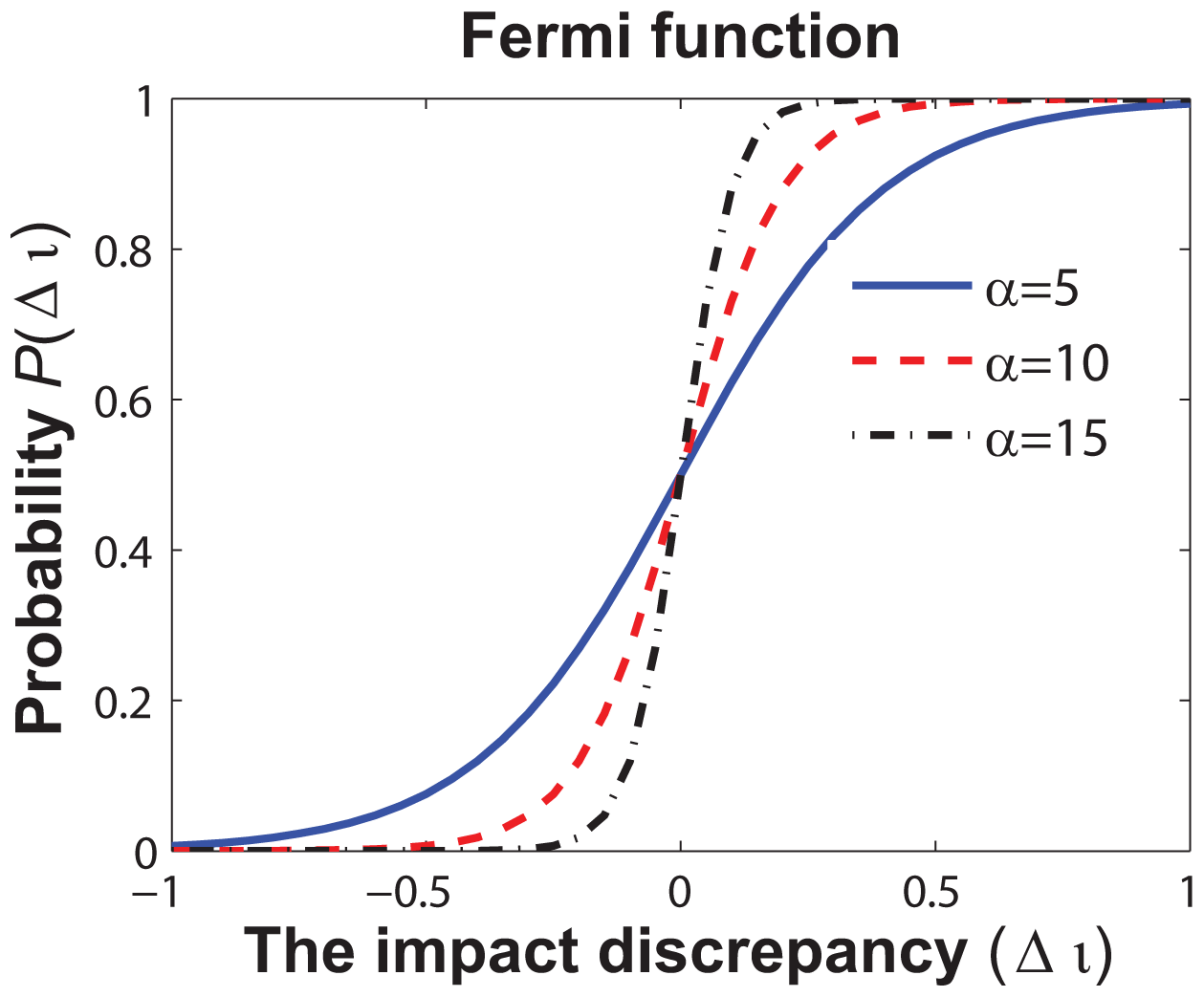
Social opinion is formalized by comparing the social influences of the two opposite opinions. For individual 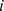, 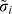 is formalized as acceptance of vaccination with the probability 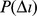 or otherwise with the probability 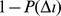. In the Fermi function, 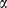 denotes individuals responsiveness to the discrepancy 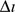.

Here, we use 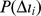 to denote the probability that social opinion 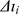 is to accept vaccination, and 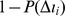 to reject vaccination. Therefore, we can write 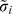 in the following form:
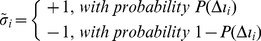
(8)where 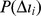 is computed from the Fermi function as follows:



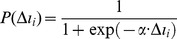
(9)The Fermi function is a sigmoid function that has been widely used for describing individuals behavioral changes as a response to the payoff discrepancy of two different choices [21], [22]. Here, 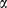 describes individuals responsiveness to the impact discrepancy of two opposite opinions. As shown in Figure 3, a larger value of 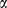 means the choice with a higher social influence will be more inclined to dominate the social opinion even the discrepancy of the two opposite social influence, 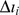, is relatively small.

Next, we introduce a probability, 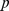, called individuals conformity rate, that indicates the degree of individuals tendency towards adopting the social opinion of his/her connected neighbors, that corresponds to how likely individual 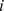 will convert his/her cost-minimized choice (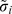) to the social influence formalized opinion (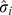). Thus, 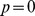 corresponds to the case of a cost-based decision maker, whereas 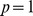 indicates that the individual is an absolute social follower (i.e., ignoring his/her own cost evaluation). In other words, the final decision of individual 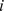 can be expressed as follows:
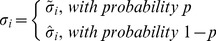
(10)


### Vaccination Threshold

In order to evaluate the impact of individuals vaccination decision making on disease control, we further construct a disease model to describe the threshold of vaccine coverage for mitigating an epidemic (i.e., the reproduction number 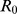 is less than one). The parameters used for estimating the vaccination threshold are listed in Table 2.

**Table 2:** Parameters used for estimating the vaccination threshold.

Symbol	Meaning
*λ*	infection risk
*β*	disease transmission rate
*γ*	recovery rate
*R*_0_	reproduction number
*θ_vac_*	vaccination threshold

For the sake of illustration, we use a standard SIR model to describe an influenza-like disease transmission in a group of individuals that are densely aggregated (e.g., students in a school), which can be treated as a homo-mixed population for disease transmission. Individuals are divided into three compartments with respect to their epidemiological states, i.e., susceptible (S), infectious (I), and recovered (R). In addition, the number of individuals in each compartment is denoted by 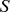, 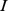, and 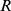. When the natural birth and death of the population are not taken into account, the overall population size is calculated as 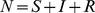. The disease spread dynamics is described by the following set of differential equations:
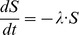


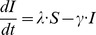
(11)

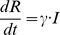
Additionally,
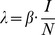
(12)where 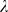 is the risk of disease infection for susceptible individuals that is proportional to the percentage of infectious population size. 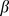 denotes the disease transmission rate that is the probability of disease transmission between the mixing of infectious and susceptible individuals. 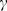 describes the recovery rate that corresponds to the time period for an infected individual to be naturally recovered and thus immunized from secondary infection.

Reproduction number 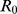 (i.e., the number of secondary infections caused by a typical infectious individual in a completely susceptible population [23], [24]) indicates a threshold for disease transmission; that is, if 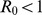, disease transmission will naturally decay. In such a compartmental disease transmission model, 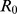 is given as follows [25], [26]:
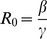
(13)


Therefore, the vaccination threshold for mitigating an epidemic is estimated as the reproduction number less than one (i.e., 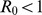). The corresponding vaccination coverage, denoted by 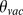, can be estimated as follows:
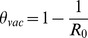
(14)


### Simulation Setting

For our simulations, we calibrate the parameters of individuals vaccination decision making based on the scenario of the 2009 H1N1 influenza epidemic, in which reproduction number 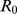 was estimated as 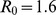 and recovery rate was set to 0.312 (i.e., a 3.2-day recovery period for disease infection) [27]–[29]. In order to focus our studies on the impact of social influence, we assume that the perceived disease transmission rate is equal to that of the actual disease transmission, i.e., 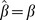. In addition, we construct a social network based on the data of individuals close proximity interactions (i.e., distance less than 3 m) at an American high school [30], where the social closeness 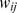 between individuals 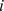 and 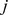 corresponds to the frequency of their interactions (i.e., the sum of all interactions between the two individuals during the day). The total number of nodes is 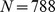 and the average node degree (i.e., the number of connected neighbors) is 35. The average edge weight (i.e., social closeness) is 115 units. Based on our model parameterization, we carry out Monte Carlo simulations to experimentally study individuals vaccination decision making and the impacts of the resulting vaccination coverage on disease control.

## Results

**Figure 4:**
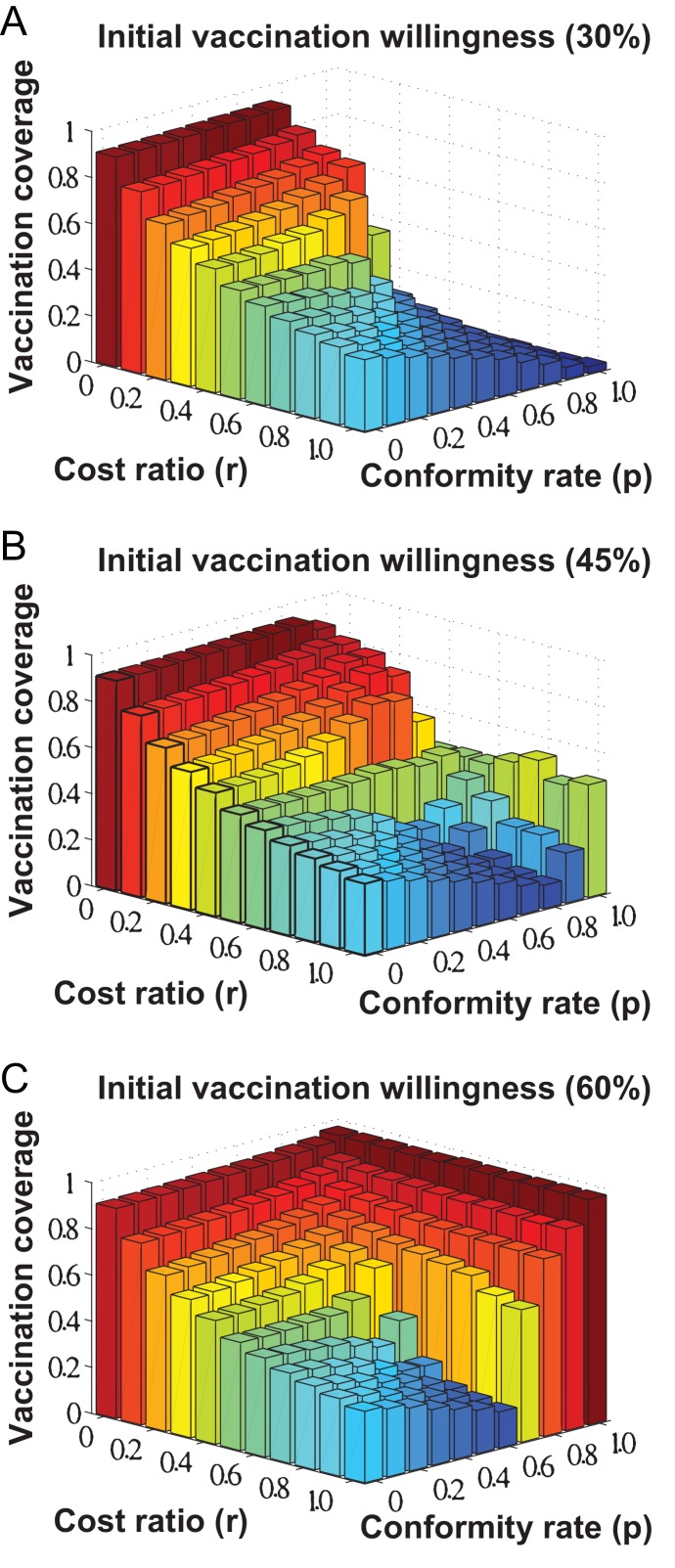
Vaccination coverage at the steady state of individuals decision making. We investigate the interplay of cost minimization and the impacts of social influence on individuals vaccination decision making, as measured by the resulting vaccination coverage, by means of varying the values of cost ratio 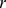 and conformity rate 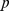 between 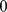 and 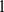. Individuals initial level of vaccination willingness is set as: (A) 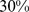; (B) 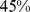; (C) 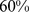.

Based on the proposed decision model, we have conducted a series of simulations on vaccination dynamics to estimate the vaccination coverage at the steady state of individuals decision making. As shown in Figure 4, we first investigate the interplay of cost minimization and the impacts of social influence on individuals vaccination decision making with reference to three initial levels of individuals vaccination willingness: 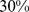, 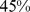, and 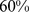. Generally speaking, the level of vaccine uptake will be subjected to the cost ratio 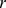 and individuals initial level of vaccination willingness, when individuals conformity rate 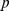 takes different values. Specifically, the simulation results in Figures 4A, 4B, and 4C show that when the impact of social influence is relative weak (i.e., conformity rate 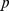 is relatively small), the cost of vaccination (i.e., cost ratio 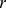) fundamentally determines the resulting vaccination coverage in that increasing the cost of vaccination will lower individuals vaccination willingness (i.e., the steady state of individuals decision making). In our considered scenario, the vaccination coverage is around 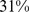 when cost ratio 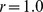. Gradually, if 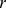 is decreased and approaches 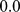, the vaccination coverage will become as high as 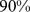. Based on our model design, when an individual perceives that all of his/her connected neighbors have decided for vaccination, the individual will keep his/her previous choice of non-vaccination even if the cost of vaccination is zero, due to the consideration that disease transmission will no longer exist.

Furthermore, we can observe that the strength of social influence (i.e., conformity rate 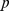) can adjust the aforementioned impacts of cost ratio 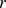 on individuals vaccination decisions. As in the extreme case that individuals are pure cost-based decision makers (i.e., 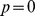), the resulting vaccination coverage will be completely determined by the relative cost of vaccination (i.e., cost ratio 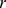). On the other hand, as in the extreme case that individuals are absolute followers of social opinion (i.e., 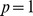), the impact of social influence will promote a universal vaccination coverage, the level of which depends on individuals initial level of willingness instead of the associated costs. In this case of simulation, when 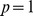, the vaccination coverage at the steady state of decision making will converge to around 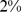 for individuals vaccination willingness at the initial level of 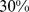 (i.e., as shown in Figure 4A), 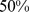 at the level of 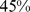 (i.e., as shown in Figure 4B), and 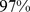 at the level of 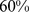 (i.e., as shown in Figure 4C).

In addition, the impacts of varying conformity rate 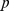 (i.e., individuals tendency to adopting social opinions) are also observed as the adjustment of vaccination decisions with reference to different situations of vaccination associated costs (i.e., cost ratio 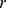). When individuals become more likely being affected by social influence (i.e., gradually increasing conformity rate 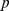), as shown in Figure 4A, the impact of social influence tends to increase the vaccination coverage when the cost of vaccination is low (i.e., 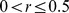). On the other hand, when the cost of vaccination is relatively high (i.e., 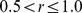), the impact of social influence will reduce the resulting vaccination coverage at the steady state of individuals decision making. Furthermore, when conformity rate 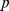 approaches 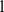, the vaccination coverage will drop/increase sharply and finally converge to a fixed level that depends on individuals initial level of vaccination willingness.

Based on the earlier-mentioned SIR model, we have investigated the impact of social influence on disease control by evaluating disease infection rates (i.e., the percentage of individuals being infected as a result of disease transmissions) with respect to different vaccination coverage resulting from individuals decision making.

**Figure 5:**
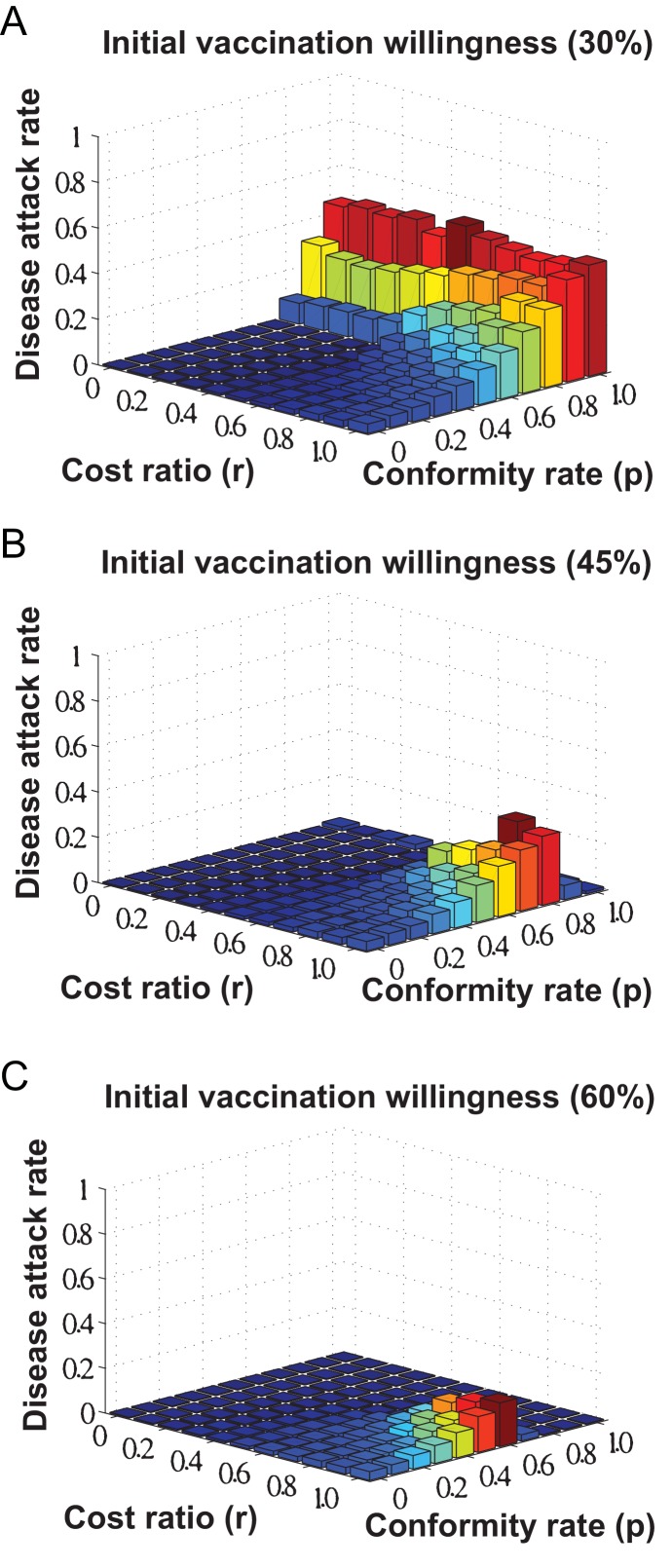
Disease attack rates with respect to different vaccination coverage resulting from individuals decision making. We investigate the interplay of cost minimization and the impacts of social influence on disease control, as measured by the percentage of individuals being infected as a result of disease transmissions, by means of varying the values of cost ratio 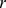 and conformity rate 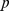 between 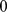 and 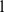. Individuals initial level of vaccination willingness is set as: (A) 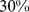; (B) 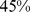; (C) 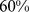.

Figure 5 shows the disease infection rates with respect to the interplay of individuals cost minimization and the impact of social influence on vaccination decision making (i.e., the values of cost ratio 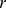 and conformity rate 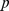 ranging between 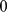 and 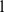, respectively). With respect to our considered epidemic scenario (i.e., basic reproduction number 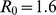), the simulation results in Figure 5A, 5B, and 5C show that disease infection can be eliminated given a relatively lower cost of vaccination (i.e., cost ratio 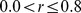) and a moderate impact of social influence (i.e., conformity rate 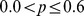).

Specifically, when individuals are less likely to being affected by social influence (i.e., conformity rate 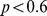), the effectiveness of disease control is generally determined by the relative cost of vaccination (i.e., cost ratio 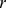) in that a lower vaccination cost can lead to a reduction in the disease infection rate due to a resulting higher vaccination coverage. Furthermore, as individuals tendency of being affected by social influence become strengthened (i.e., conformity rate 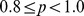), the effect of vaccination cost on disease control will be weakened accordingly, while individuals initial level of vaccination willingness matters. In the extreme case of 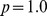 (i.e., individuals are absolute followers of social influence), the disease infection rate is observed as high as 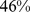 for the initial level of vaccination willingness at 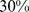, as shown in Figure 5A. If the initial level of vaccination willingness is set as 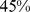 (i.e., as shown in Figure 5B), the disease attack rate will be relatively higher than the situation of the initial level at 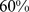 (i.e., as shown in Figure 5C), where cost ratio 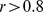 and conformity rate 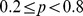.

**Figure 6:**
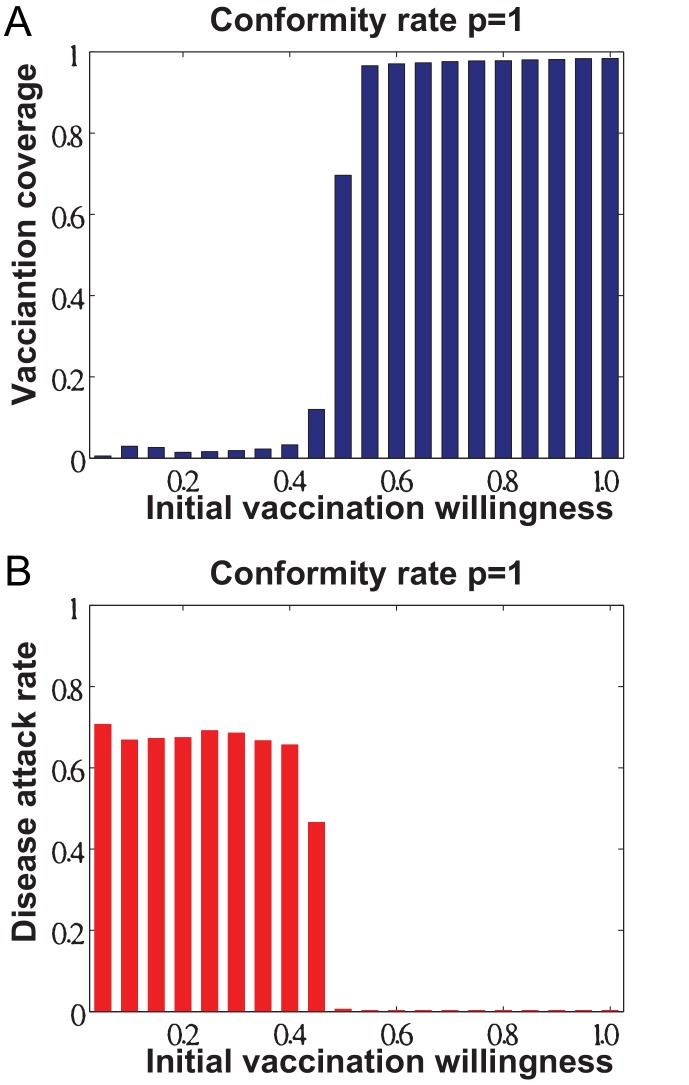
The impacts of individuals initial levels of vaccination willingness when individuals are all social followers (i.e., conformity rate *p*  =  1.0). (A) vaccination coverage at the steady state of individuals decision making. (B) the resulting effects on epidemic control in terms of disease attack rate.

Besides, we have examined the steady-state vaccination coverage and the resulting disease attack rate with respect to different initial levels of individuals vaccination willingness prior to their decision making, the results of which are shown in Figure 6. We can note that individuals initial level of willingness will affect the converged level of the steady-state vaccination coverage as well as the effectiveness of disease control when individuals are absolute followers of social opinions (i.e., conformity rate 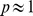). In our simulations, when the initial level of individuals vaccination willingness is 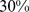, the converged steady-state vaccination coverage is around 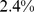 (Figure 6A). The vaccination coverage will reach 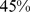 and 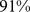, if the initial levels of vaccination willingness are 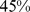 and 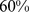, respectively. In addition, we can observe that there exists a critical phase transition in vaccination coverage when individuals initial level of vaccination willingness is between 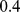 and 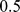 (Figure 6A). That is to say, in the situation of individuals being absolute social followers, there is a threshold value in terms of individuals initial level of vaccination willingness that can be used to evaluate the effectiveness of a voluntary vaccination program for eliminating the epidemic (Figure 6B).

### Sensitivity Analysis

**Figure 7:**
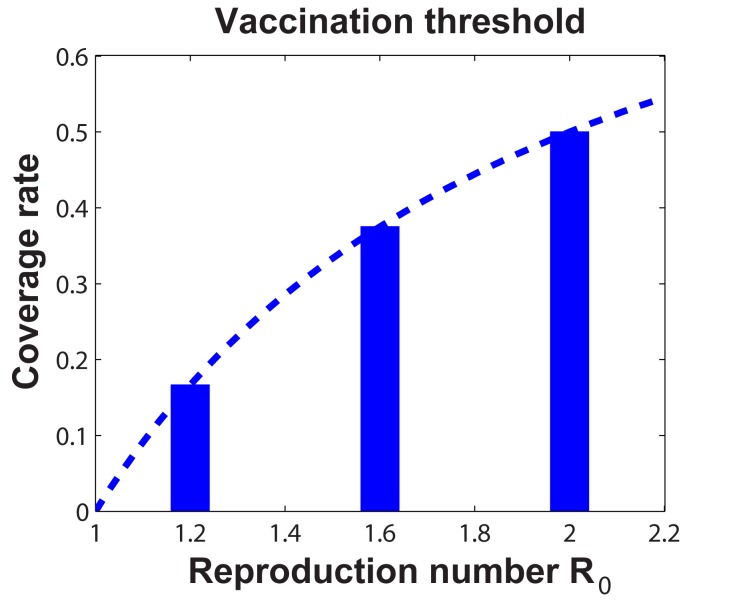
The vaccination thresholds for eliminating the epidemic with respect to different disease reproduction numbers: (1) *R*_0_  =  1.2; (2) *R*_0_  =  1.6; (3) *R*_0_  =  2.0.

In order to investigate the sensitivity of our results, in what follows, we further consider individuals vaccination decision making with respect to the different values of disease reproduction number: (1) 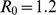; (2) 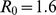; (3) 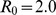. Figure 7 shows the vaccination thresholds for eliminating the epidemic with respect to different basic reproduction numbers 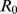.

**Figure 8:**
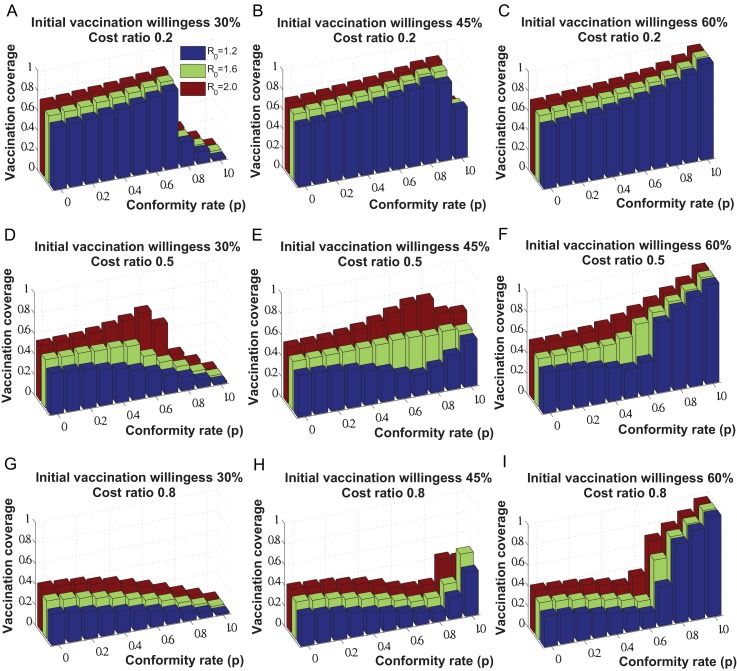
Sensitivity analysis of the vaccination coverage at the steady state of individuals decision making with respect to the different values of disease reproduction number: (1) *R*_0_  =  1.2; (2) *R*_0_  =  1.6; (3) *R*_0_  =  2.0.

Figure 8 shows the vaccination coverage at the steady state of individuals decision making with respect to different disease reproduction numbers. Here, we can observe the similar impacts of social influence in all three considered situations: the impact of social influence will increase the vaccination coverage when the relative cost of vaccination 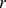 is small (see Figures 8A, 8B, and 8C), decrease it when 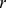 is relatively large (see Figures 8.g, 8.h, and 8.i), and bring it to a certain level when individuals become followers of social influence (i.e., conformity rate 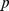 approaches 1). The simulation results further show that when the impact of social influence is relatively weak (i.e., conformity rate 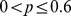), relatively severe disease transmissions in terms of a larger reproduction number (i.e., 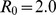) will increase the vaccination coverage. While, if the impact of social influence is strengthened (i.e., conformity rate 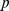 approaches 1), the vaccination coverage at the steady state of individuals decision making is mostly determined by individuals initial level of vaccination willingness, rather than the related costs and disease severity.

## Discussion

The phenomena of social influence that individuals behaviors or opinions are affected by their social environment have long been observed and studied, such as in the domains of political voting [31], [32] and consumer purchasing decisions [33], [34]. In the context of vaccination, social influence can affect individuals vaccination decisions and thus the effectiveness of disease control in terms of the resulting vaccination coverage [10]. In this study, we address the impact of social influence on individuals vaccination decision making, vaccination coverage, and disease control. Towards this end, we have provided a dual-perspective view on modeling individuals vaccination decision making by incorporating the impact of social influence with the game-theoretical analysis of vaccination cost minimization. In a group of individuals, the impact of social influence on an individuals decision making relies on the structure of how he/she interacts with others. In order to characterize the impact of social influence in such an interactive environment, we have used social impact theory (SIT) to characterize the strength of social influence on changing individuals vaccination decisions with respect to their interaction relationships. We have used individuals social network to represent the structure of their interaction relationships. Based on our proposed model, we have examined the impact of social influence on individuals decisions and on the effectiveness of disease control (i.e., vaccination coverage), with respect to three determinants: (1) the relative cost of vaccination decision, i.e., cost ratio 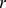; (2) individuals conformity to social influence, i.e., conformity rate 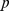; and (3) individuals initial level of vaccination willingness.

By parameterizing the proposed model with a real-world contact network and with the epidemiological scenario of 2009 H1N1 influenza, we have carried out a series of simulations on individuals voluntary vaccination. The simulation results have confirmed that the relative cost of vaccination (i.e., cost ratio 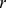) is one of the determining factors in the voluntary vaccination coverage. In our simulations, such results can be observed if individuals are less likely to be affected by social influence (i.e., conformity rate 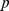 is relatively small). While, if individuals become more susceptible to social influence (i.e., 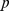 is large), the impact of social influence has been found to increase the vaccination coverage when the cost of vaccination is small and, conversely, reduce the vaccination coverage when the cost is large. In the extreme case where individuals are absolute social followers (i.e., conformity rate 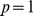), the vaccination coverage at the steady state would converge to a certain level that merely depends on individuals initial level of vaccination willingness, instead of the vaccination associated costs.

In modeling individuals vaccination decision making, several mathematical models have been earlier proposed that utilize payoff-based approaches to characterizing vaccination decision making with respect to individuals perceived costs and benefits of vaccination [35]–[37]. Bauch et al. [38], [39] characterized individuals vaccination decisions as a modified minority game by exploring the herd immunity effect; that is, in a group of mixed individuals, vaccinating a proportion of them would decrease the infection risk for the rest of individuals [40]. In consideration of that, game theory has been used to describe individuals interactive decision making in favor of optimizing personal payoffs [41], [42]. Cojocaru [43] extended the game-theoretical model of vaccination decision making by considering a finite number of heterogeneous population groups. Perisic et al. [44], [45] further incorporated individuals contact networks into the vaccination game analysis. Moreover, some studies have considered social and psychological aspects of decision making (e.g., social learning process [46] and imitation behaviors [21], [22], [47], [48]). While, others have considered the issues of incomplete information by adding either the potential discrepancy between individuals perceptions and real situations (e.g., the perceived disease prevalence and the adverse effects of vaccine [49], [50]) or different sources of information (e.g., previous disease prevalence or vaccination programs [51]–[53]). Besides the payoff-based analysis, Salathe et al. in [54] investigated the clustering of vaccinated and unvaccinated individuals with an opinion formation model. They proposed that the probability for an individual changing his/her vaccination opinion is proportional to the ratio of neighbors that have an opposite opinion.

As an improvement over the above-mentioned existing models, we consider an individuals vaccination decision as a hybrid process balancing his/her self-initiated cost minimization (i.e., individuals minority-seeking-like behaviors by exploring the herd immunity effect) as well as the social influence of neighbors decisions (i.e., social conformity behaviors). Our model introduces a parameter 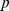 (i.e., conformity rate) to modulate individuals tendency towards these two decision making mechanisms: an individual will adopt his/her cost-minimized decision, or convert to the social opinion of his/her connected neighbors. Different from the existing studies that address individuals vaccination decision making as a process of opinion formation (e.g., Salathe et al. in [54]), here we further take into account the heterogeneities of individuals interaction relationships by exploiting an extended SIT-based characterization of the strength of social influence. Additionally, by incorporating the impact of social influence, we are able to investigate the impact of individuals initial level of vaccination willingness on the vaccination coverage of individuals decision making.

By computationally characterizing the impact of social influence, this study has practical implications for understanding individuals vaccination behaviors and for improving the effectiveness of adopted vaccination policies. In the recent years, the rapidly increasing use of new communication tools e.g., internet-based social media services, has further amplified such a social influence [55]–[58]. For instance, the efficacy or the adverse effects of vaccines would be debated [59], [60], and the opinions on either accepting or rejecting vaccination would fast spread among individuals [61], [62]. We have identified that individuals initial level of vaccination willingness as an important factor in determining the final vaccination coverage due to the impact of social influence (i.e., individuals social conformity). Our results have shown that when conformity rate 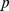 approaches 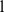, the vaccination coverage at the steady state of individuals decision making will be polarized given different initial levels of individuals vaccination willingness. Moreover, the empirical studies that survey the determinants of individuals vaccination decisions in a social environment can readily provide us a practical means for measuring and evaluating individuals conformity to social influence [63]. As has been shown in our study that individuals vaccination decisions can be affected by both the associated costs and their conformity to social influence, it becomes necessary and feasible for public health authorities to estimate the level of individuals acceptance of vaccine prior to the start of a voluntary vaccination program, as well as to timely assess and enhance the effectiveness of their adopted vaccination policies, e.g., providing certain financial subsidies to reduce the cost of vaccination.

So far, our study has provided a general modeling framework for incorporating the impact of social influence into the individuals decision making and disease control. It should be pointed out that the obtained results of this study may be subjected to the considered social network (e.g., students interactions within an American high school). In our proposed model, the social influence accounts only for the localized interactions between an individual with his/her connected neighbors. Additionally, by utilizing the SIT-based characterization of social impact, an implicit assumption is that individuals are passive recipients of social influence and their active behaviors have not been taken into account.

It would be interesting for us to further consider some of the related aspects in our future work:

### 

#### 1. The effects of public media

Public media represents another type of information source that will affect individuals vaccination decision making. Due to broadcasting effect, the transmission of social influence through public media may be faster and wider. Related work by Breban [15] discussed the effects of media on the fluctuation of vaccination coverage. For the future work, it is possible to extend the current model by incorporating the effects of public media, e.g., by adding a super node that interacts with a large portion of nodes.

#### 2. Host population heterogeneity

To focus on the SIT-based characterization of social influence, we have assumed that individuals are homogeneous in disease infection, e.g., susceptibility, infectivity, and infection risk. In this regard, our modeling framework will be further extended to incorporating individual variations in disease transmission as well as in their social characteristics (e.g., creditability). It would be desirable to further improve our simulations by differentiating physical contacts for infectious disease transmission from interaction relationships for social influence. Along this line, related work by Eames [16] constructed a parent network for describing vaccination decision and a children network for representing disease transmission, and found that the impact of social influence would be influenced by the overlap of these two networks.

#### 3. Dynamics of disease spread

In this work, we have only considered individuals making vaccination decisions based on their perceived infection risk, which may come from either their previous experience of disease and vaccine or the awareness about the upcoming epidemic season. In the real world, real-time disease dynamics could also affect vaccination dynamics, i.e., disease outbreaks may increase individuals willingness for vaccination. In the future, we will extend our model by characterizing the interplays between individuals vaccination decisions and the dynamics of disease spread.

## References

[1] Galvani AP, Reluga TC, Chapman GB (2007) Long-standing influenza vaccination policy is in accord with individual self-interest but not with the utilitarian optimum. Proc Natl Acad Sci 104(13): 5692–5697. 17369367 10.1073/pnas.0606774104 PMC1838447

[2] Wu B, Fu F, Wang L (2011) Imperfect vaccine aggravates the long-standing dilemma of voluntary vaccination. PLoS ONE 6(6): e20577. 21687680 10.1371/journal.pone.0020577 PMC3110791

[3] Fine P, Eames K, Heymann DL (2011) “Herd immunity”: a rough guide. Clin Infect Dis 52(7): 911–916. 21427399 10.1093/cid/cir007

[4] Myers LB, Goodwin R (2011) Determinants of adults intention to vaccinate against pandemic swine flu. BMC Public Health 11(1): 11–15. 21211000 10.1186/1471-2458-11-15 PMC3024930

[5] Eastwood K, Durrheim DN, Jones A, Butler M (2010) Acceptance of pandemic (H1N1) 2009 influenza vaccination by the Australian public. Med J Aust 192(1): 33–36. 20047546 10.5694/j.1326-5377.2010.tb03399.x

[6] Liao Q, Cowling BJ, Lam WWT, Fielding R (2011) Factors affecting intention to receive and self-reported receipt of 2009 pandemic (H1N1) vaccine in Hong Kong: a longitudinal study. PLoS ONE 6(3): e17713. 21412418 10.1371/journal.pone.0017713 PMC3055876

[7] Streefland PH (2001) Public doubts about vaccination safety and resistance against vaccination. Health Policy 55(3): 159–172. 11164965 10.1016/s0168-8510(00)00132-9

[8] Francois G, Duclos PD, Margolis H, Lavanchy D, Siegrist CA, et al (2005) Vaccine safety controversies and the future of vaccination programs. Pediatr Infect Dis J 24(11): 953–961. 16282928 10.1097/01.inf.0000183853.16113.a6

[9] Lau JTF, Yeung NCY, Choi KC, Cheng MYM, Tsui HY, et al.. (2009) Acceptability of A/H1N1 vaccination during pandemic phase of influenza A/H1N1 in Hong Kong: population based cross sectional survey. BMJ 339(b4164). 10.1136/bmj.b4164 PMC2768779 19861377

[10] Larson HJ, Cooper LZ, Eskola J, Katz SL, Ratzan S (2011) Addressing the vaccine confidence gap. Lancet 378(9790): 526–535. 21664679 10.1016/S0140-6736(11)60678-8

[11] Lau JTF, Yeung NCY, Choi KC, Cheng MYM, Tsui HY, et al (2010) Factors in association with acceptability of A/H1N1 vaccination during the influenza A/H1N1 pandemic phase in the Hong Kong general population. Vaccine 28(29): 4632–4637. 20457289 10.1016/j.vaccine.2010.04.076 PMC7131323

[12] Zijtregtop EAM, Wilschut J, Koelma N, Delden JJMV, Stolk RP, et al (2009) Which factors are important in adults uptake of a (pre)pandemic influenza vaccine? Vaccine 28(1): 207–227. 19800997 10.1016/j.vaccine.2009.09.099

[13] Barriere J, Vanjak D, Kriegel I, Otto J, Peyrade F, et al (2010) Acceptance of the 2009 A(H1N1) influenza vaccine among hospital workers in two French cancer centers. Vaccine 28(43): 7030–7034. 20817011 10.1016/j.vaccine.2010.08.021

[14] Tai Z, Sun T (2007) Media dependencies in a changing media environment: the case of the 2003 SARS epidemic in China. New Media Soc 9(6): 987–1009.

[15] Breban R (2011) Health newscasts for increasing influenza vaccination coverage: an inductive reasoning game approach. PLoS ONE 6(12): e28300. 22205944 10.1371/journal.pone.0028300 PMC3244398

[16] Eames KTD (2009) Networks of influence and infection: parental choices and childhood disease. J R Soc Interface 6(38): 811–814. 19447820 10.1098/rsif.2009.0085 PMC2820361

[17] Watts DJ, Dodds PS (2007) Influentials, networks, and public opinion formation. J Consum Res 34(4): 441–458.

[18] Latane B (1981) The psychology of social impact. Am Psychol 36(4): 343–356.

[19] Nowak A, Szamrej J, Latane B (1990) From private attitude to public opinion: A dynamic theory of social impact. Psychol Rev 97(3): 362–376.

[20] Holys JA, Schweitzer F (2001) Social impact models of opinion dynamics. Annu Rev Comput Phys 9: 253–272.

[21] Fu F, Rosenbloom DI, Wang L, Nowak MA (2011) Imitation dynamics of vaccination behaviour on social networks. Proc R Soc B 278(1702): 42–49. 10.1098/rspb.2010.1107 PMC2992723 20667876

[22] Mbah MLN, Liu J, Bauch CT, Tekel YI, Medlock J, et al (2012) The impact of imitation on vaccination behavior in social contact networks. PLoS Comput Biol 8(4): e1002469. 22511859 10.1371/journal.pcbi.1002469 PMC3325186

[23] Heesterbeek JAP (2002) A brief history of R0 and a recipe for its calculation. Acta Biotheor 50(3): 189–204. 12211331 10.1023/a:1016599411804

[24] Heffernan JM, Smith RJ, Wahl LM (2005) Perspectives on the basic reproductive ratio. J R Soc Interface 2(4): 281–293. 16849186 10.1098/rsif.2005.0042 PMC1578275

[25] Keeling MJ, Grenfell BT (2000) Individual-based perspectives on R0. J theor Biol 203(1): 51–61. 10677276 10.1006/jtbi.1999.1064

[26] Diekmann O, Heesterbeek JAP (2000) Mathematical epidemiology of infectious diseases: model building, analysis and interpretation. Wiley.

[27] Yang Y, Sugimoto JD, Halloran E, Basta NE, Chao DL, et al (2009) The transmissibility and control of pandemic influenza A (H1N1) virus. Science 326(5953): 729–733. 19745114 10.1126/science.1177373 PMC2880578

[28] Fraser C, Donnelly CA, Cauchemez S, Hanage WP, Kerkhove MDV, et al (2009) Pandemic potential of a strain of influenza A (H1N1): early findings. Science 324: 1557–1561. 19433588 10.1126/science.1176062 PMC3735127

[29] Cowling BJ, Lau MSY, Ho LM, Chuang SK, Tsang T, et al (2010) The effective reproduction number of pandemic influenza: prospective estimation. Epidemiology 21(6): 842–846. 20805752 10.1097/EDE.0b013e3181f20977 PMC3084966

[30] Salathe M, Kazandjieva M, Lee JW, Levis P, Feldman MW, et al (2010) A highresolution human contact network for infectious disease transmission. Proc Natl Acad Sci 107(51): 22020–22025. 21149721 10.1073/pnas.1009094108 PMC3009790

[31] Kottonau J, Pahl-Wostl C (2004) Simulating political attitudes and voting behavior. Journal of Artificial Societies and Social Simulation 7(4): 107–108.

[32] Singh VK, Basak S, Modanwal N (2011) Agent based modeling of individual voting preferences with social influence. In: Trends in Computer Science, Engineering and Information Technology. vol. 204 of Communications in Computer and Information Science. Springer Berlin Heidelberg; p. 542–552.

[33] Burnkrant RE, Cousineau A (1975) Informational and normative social influence in buyer behavior. J Consum Res 2(3): 206–215.

[34] Grinblatt M, Keloharju M, Ikaheimo S (2008) Social influence and consumption: evidence from the automobile purchases of neighbors. Rev Econ Stat 90(4): 735–753.

[35] Chen FH (2006) A susceptible-infected epidemic model with voluntary vaccinations. J Math Biol 53: 253–272. 16758209 10.1007/s00285-006-0006-1

[36] Codeco CT, Luz PM, Coelho F, Galvani AP, Struchiner C (2006) Vaccinating in disease-free regions: a vaccine model with application to yellow fever. J R Soc Interface 4(17): 1119–1125. 10.1098/rsif.2007.0234 PMC2396207 17442650

[37] Vardavas R, Breban R, Blower S (2007) Can Influenza Epidemics Be Prevented by Voluntary Vaccination? PLoS Comput Biol 3(5): e85. 17480117 10.1371/journal.pcbi.0030085 PMC1864996

[38] Bauch CT, Galvani AP, Earn DJD (2003) Group interest versus self-interest in smallpox vaccination policy. Proc Natl Acad Sci 100(18): 10564–10567. 12920181 10.1073/pnas.1731324100 PMC193525

[39] Bauch CT, Earn DJD (2004) Vaccination and the theory of games. Proc Natl Acad Sci 101(36): 13391–13394. 15329411 10.1073/pnas.0403823101 PMC516577

[40] John TJ, Samuel R (2000) Herd immunity and herd effect: new insights and definitions. Eur J Epidemiol 16(7): 601–606. 11078115 10.1023/a:1007626510002

[41] Reluga TC, Bauch CT, Galvani AP (2006) Evolving public perceptions and stability in vaccine uptake. Math Biosci 204(2): 185–198. 17056073 10.1016/j.mbs.2006.08.015

[42] Reluga TC, Galvani AP (2011) A general approach for population games with application to vaccination. Math Biosci 230(2): 67–78. 21277314 10.1016/j.mbs.2011.01.003 PMC3063328

[43] Cojocaru MG (2008) Dynamic equilibria of group vaccination strategies in a heterogeneous population. J Glob Optim 40: 51–63.

[44] Perisic A, Bauch CT (2009) Social contact networks and disease eradicability under voluntary vaccination. PLoS Comput Biol 5(2): e1000280. 19197342 10.1371/journal.pcbi.1000280 PMC2625434

[45] Perisic A, Bauch CT (2009) A simulation analysis to characterize the dynamics of vaccinating behaviour on contact networks. BMC Infect Dis 9(1): 77. 19476616 10.1186/1471-2334-9-77 PMC2695470

[46] Bauch CT, Bhattacharyya S (2012) Evolutionary game theory and social learning can determine how vaccine scares unfold. PLoS Comput Biol 8(4): e1002452. 22496631 10.1371/journal.pcbi.1002452 PMC3320575

[47] Bauch CT (2005) Imitation dynamics predict vaccinating behaviour. Proc R Soc B 272(1573): 1669–1675. 10.1098/rspb.2005.3153 PMC1560180 16087421

[48] dOnofrio A, Manfredi P, Poletti P (2011) The impact of vaccine side effects on the natural history of immunization programmes: an imitation-game approach. J Theor Biol 273(1): 63–71. 21187103 10.1016/j.jtbi.2010.12.029

[49] Coelho FC, Codeco CT (2009) Dynamic modeling of vaccinating behavior as a function of individual beliefs. PLoS Comput Biol 5(7): e1000425. 19593365 10.1371/journal.pcbi.1000425 PMC2700262

[50] Zhang H, Zhang J, Li P, Small M, Wang B (2011) Risk estimation of infectious diseases determines the effectiveness of the control strategy. Physica D 240(11): 943–948. 10.1016/j.physd.2011.02.001 PMC7114255 32287556

[51] dOnofrio A, Manfredi P, Salinelli E (2007) Vaccinating behaviour, information, and the dynamics of SIR vaccine preventable diseases. Theor Popul Biol 71(3): 301–317. 17335862 10.1016/j.tpb.2007.01.001

[52] Breban R, Vardavas R, Blower S (2007) Mean-field analysis of an inductive reasoning game: Application to influenza vaccination. Phys Rev E 76(3): 031127. 10.1103/PhysRevE.76.031127 17930219

[53] dOnofrio A, Manfredi P (2010) Vaccine demand driven by vaccine side effects: dynamic implications for SIR diseases. J Theor Biol 264(2): 237–252. 20149801 10.1016/j.jtbi.2010.02.007

[54] Salathe M, Bonhoeffer S (2008) The effect of opinion clustering on disease outbreaks. J R Soc Interface 5(29): 1505–1508. 18713723 10.1098/rsif.2008.0271 PMC2607358

[55] Keelan J, Pavri-Garcia V, Tomlinson G, Wilson K (2007) YouTube as a source of information on immunization: a content analysis. JAMA 298(21): 2482–2484. 18056901 10.1001/jama.298.21.2482

[56] Vance K, Howe W, Dellavalle RP (2009) Social internet sites as a source of public health information. Dermatol Clin 27(2): 133–136. 19254656 10.1016/j.det.2008.11.010

[57] Pandey A, Patni N, Singh M, Sood A, Singh G (2010) YouTube as a source of information on the H1N1 influenza pandemic. Am J Prev Med 38(3): e1–e3. 20171526 10.1016/j.amepre.2009.11.007

[58] Signorini A, Segre AM, Polgreen PM (2011) The use of Twitter to track levels of disease activity and public concern in the US during the influenza A H1N1 pandemic. PLoS ONE 6(5): e19467. 21573238 10.1371/journal.pone.0019467 PMC3087759

[59] Keelan J, Pavri V, Balakrishnan R, Wilson K (2010) An analysis of the Human Papilloma Virus vaccine debate on MySpace blogs. Vaccine 28(6): 1535–1540. 20003922 10.1016/j.vaccine.2009.11.060

[60] Witteman HO, Zikmund-Fisher BJ (2012) The defining characteristics of Web 2.0 and their potential influence in the online vaccination debate. Vaccine 30(25): 3734–3740. 22178516 10.1016/j.vaccine.2011.12.039

[61] Henrich N, Holmes B (2011) What the public was saying about the H1N1 vaccine: perceptions and issues discussed in on-line comments during the 2009 H1N1 pandemic. PLoS ONE 6(4): e18479. 21533161 10.1371/journal.pone.0018479 PMC3078916

[62] Salathe M, Khandelwal S (2011) Assessing vaccination sentiments with online social media: implications for infectious disease dynamics and control. PLoS Comput Biol 7(10): e1002199. 22022249 10.1371/journal.pcbi.1002199 PMC3192813

[63] Bish A, Yardley L, Nicoll A, Michie S (2011) Factors associated with uptake of vaccination against pandemic influenza: A systematic review. Vaccine 29(38): 6472–6484. 21756960 10.1016/j.vaccine.2011.06.107

